# Three-metal-ion catalysis by ribonuclease P holoenzyme: a new mechanistic insight into transfer RNA maturation

**DOI:** 10.21203/rs.3.rs-9187222/v1

**Published:** 2026-05-14

**Authors:** Yun-Tzai Lee, Maximilia F. S. Degenhardt, Ilias Skeparnias, Szu-Yun Chen, Bapurao A. Bhoge, Sergey G. Tarasov, Marzena A. Dyba, Dan Shi, Jinwei Zhang, Jason R. Stagno, Yun-Xing Wang

**Affiliations:** 1 Protein-Nucleic Acid Interaction Section, Center for Structural Biology, National Cancer Institute, Frederick, Maryland 21702, USA; 2 Laboratory of Molecular Biology, National Institute of Diabetes and Digestive and Kidney Diseases, Bethesda, MD 20892, USA; 3 Biophysics Resource, Center for Structural Biology, National Cancer Institute, Frederick, Maryland 21702, USA; 4 Cryo-EM Facility, Center for Structural Biology, National Cancer Institute, Frederick, Maryland 21702, USA

## Abstract

Ribonuclease P (RNase P) is an essential metalloenzyme responsible for tRNA 5′ maturation across all three domains of life. The catalytic mechanism, particularly the number of divalent metal ions and the role of its protein component, remains unresolved. We determined cryo-EM structures of *Geobacillus Stearothermophilus* RNase P holoenzyme at 2.7-3.0 Å resolution in three catalytic states. These structures reveal a three-metal-ion catalytic mechanism, challenging the long-standing two-metal-ion model. Two metal ions are substrate-dependent, one of which is also protein-dependent. The protein component, rnpA, dramatically enhances ribozyme activity by reducing conformational sampling, stabilizing the enzyme-substrate complex through direct contact with the pre-tRNA 5′-leader, and cooperatively stabilizing the third catalytic metal ion with the substrate.

Ribonuclease P (RNase P) is an essential endoribonuclease for the 5′ maturation of transfer RNAs (tRNAs) across all three domains of life. In bacteria and some archaea, the RNA component of RNase P (RPR), known as the ribozyme (apo-state), exhibits intrinsic catalytic activity *in vitro*, although it is extremely inefficient compared to RNase P holoenzyme (holo-state), whose activity is enhanced by a single protein component^1,2^. In contrast, the eukaryotic RPRs lack independent ribozyme activity and require ~9-10 protein subunits as a scaffold to form a large ribonucleoprotein (RNP) complex for substrate recognition and catalysis, both *in vitro* and *in vivo*^3,4^.

The bacterial RNase P requires a single protein, RNase P protein A (rnpA), which enhances substrate-binding affinity and enzymatic efficiency by approximately 180-fold^5–10^. Yet little is known about how rnpA achieves this and its role throughout the catalytic cycle, because the structures of apo and holo RNase P in different reaction states have not been fully determined at a resolution sufficient to elucidate the enzymatic mechanism. Such information is crucial for understanding the structural basis of catalysis at each stage of a complete enzymatic cycle, specifically how the protein component enhances substrate-binding affinity, cleavage efficiency, and product release by altering the structure of RPR and the interaction modes with the substrate and metal ions.

The critical roles of Mg^2+^ ions are widely known for the stabilization of the RPR structure^11,12^. However, there is a lack of definitive structural evidence to support the long-proposed two-metal-ion mechanism for RNase P catalysis^13,14^. Previously reported bacterial RNase P structures were determined at relatively low resolution^15–19^, none of which are sufficient to accurately elucidate the structural details of catalytic metal ions, let alone in different reaction states. Thus, the exact number and precise coordination of metal ions in the catalytic center of RNase P are still unknown. In this work, we report a series of high-resolution structures that provide direct structural evidence of three coordinated metal ions in the RNase P catalytic core. One of these ions, recruited by the precursor tRNA (pre-tRNA) substrate and further stabilized by RNase P protein component, is observed only when both substrate and rnpA are bound. The third metal ion has thus far been observed in several classes of DNA polymerases and facilitates the formation of the phosphodiester bond or stabilizes the product^20–22^. Our results show that the RNase P holoenzyme uses an analogous three-metal-ion scheme to catalyze the cleavage of a phosphodiester bond. These findings provide mechanistic and evolutionary insights into how an ancient, *trans*-acting ribozyme gradually acquired protein cofactor(s) to restrict RNA conformational sampling, facilitate substrate binding and placement, and stabilize its catalytic configuration.

## Results

### Structures of RNase P holoenzyme in a complete catalytic cycle

**Fig. 1 |:**
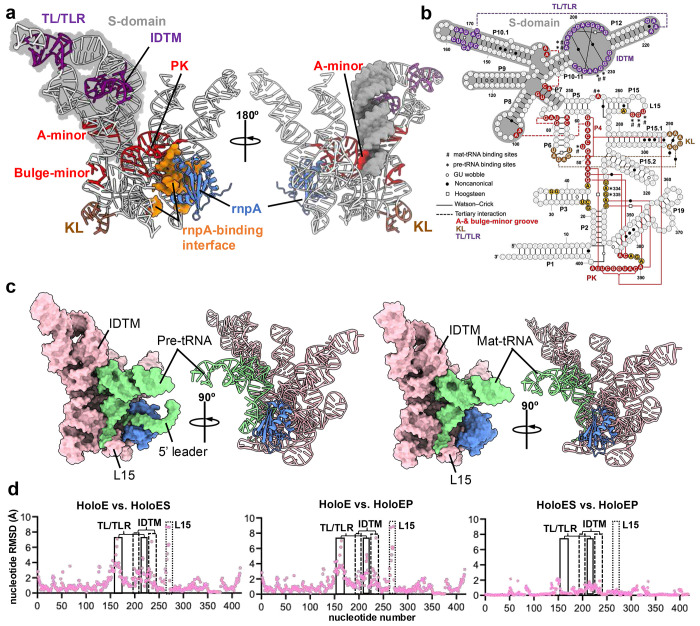
Structures of RNase P holoenzyme (holoE) and in complex with pre-tRNA (holoES) and mat-tRNA (holoEP). **a**, Cryo-EM all-atom structure of holoE with all tertiary interactions and rnpA-binding region (orange surface) shown. **b**, The detailed secondary structure holoE, with the same color scheme mapping as in the three-dimensional structure shown in (a). **c**, Cryo-EM all-atom structures of holoES (left) and holoEP (right). The two regions, IDTM and L15 of RPR, that undergo conformational changes upon binding to pre-tRNA substrate, are indicated. **d**, Per-nucleotide root-mean-square-difference (RMSD) between holoE and holoES (left), holoE and holoEP (middle), and holoES and holoEP (right).

Using cryo-EM, we determined and systematically analyzed eight structures of *Bacillus*-type (B-type) RNase P holoenzyme (holoE) from *Geobacillus stearothermophilus* (*Gst* RNase P) under divalent cation conditions containing 1 or 5 mM Ca^2+^ or Mg^2+^, and in complex with precursor tRNAs (pre-tRNAs) or mature tRNA (mat-tRNA) (**Extended Data Figs. 1 and 2**). At the resolution (2.7-3.0 Å), these structures enable accurate mapping of detailed secondary structures and long-range tertiary interactions (Fig. 1a and 1b). The three primary structures that illustrate the full catalytic cycle include the holoenzyme alone (holoE), its complexes with either its cognate pre-tRNA^Gly^ substrate (holoES) and mat-tRNA product (holoEP) at 5 mM Ca^2+^ (Fig. 1a and 1c, **Supplementary Table 1, and Extended Data Figs. 1**). These three structures indicate that the RPR maintains the overall fold throughout the reaction cycle. The catalytic (C) domain of RPR in each structure is well-defined at a local resolution of ~2.5 Å (**Extended Data Fig. 1a**). The substrate-specificity (S) domain, however, shows greater mobility and lower local resolution of ~5 Å (**Extended Data Fig. 1a**), consistent with its dynamic motions described previously^12^. However, the local resolution in the lower segment of the S-domain is markedly improved to ~4 Å in the presence of tRNA (**Extended Data Fig. 1a**), suggesting that the substrate stabilizes this otherwise flexible domain. Importantly, the cryo-EM volumes were complete and adequate for atomic modeling of the entire S-domain (**Extended Data Fig. 1b**). Among the three enzymatic-state structures, several long-range tertiary interactions within RPR are observed, such as the kissing loop (KL), tetraloop/tetraloop receptor (TL/TLR), bulge-minor groove, and A-minor groove interactions (Fig. 1a and 1b). One of these A-minor groove interactions, involving two consecutive adenine bases (A99 and A100) in the S-domain and the minor groove of P4 in the C-domain, is the only inter-domain interaction (Fig. 1a and 1b). This interaction was also observed previously in some RPR conformers at 1 mM Mg^2+^, suggesting its transient nature in the absence of substrate^12^. The most notable structural changes induced by pre-tRNA or mat-tRNA binding in RPR are observed in loop 15 (L15) and the interdigitated double T-loop motif (IDTM)^23–26^ in the S-domain, due to the direct contact with tRNA (Fig. 1c and 1d).

### RnpA binding and its impact on RNase P RNA stability

**Fig. 2 |:**
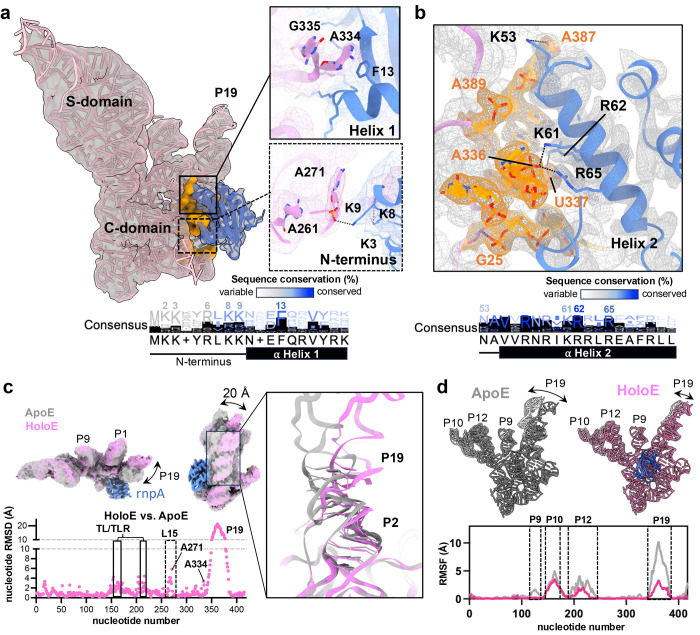
Interaction with rnpA and its stabilizing effect on RPR. **a**, RNase P holoenzyme (holoE) structure, including RNase P RNA (RPR) and rnpA, colored in pink and blue, respectively, with the RPR nucleotides in the rnpA-binding interface rendered as an orange surface. Inset panels: detailed interactions between RPR and rnpA residues in α helix 1 (top) and the amino-terminus (bottom). Sequence conservation for this region of rnpA is shown. **b**, Same as in (a) but for interactions involving rnpA Helix 2. **c**, Structural comparison of RPR-alone (apoE, gray) and holoE (pink), showing the significant structural changes to one of the peripheral structural elements, P19 (right panel), caused by rnpA binding. **d**, RnpA binding reduces structural heterogeneity of the structural elements in the substrate-specificity (S-) domain (P9-10 and P12) and P19 in the catalytic (C-) domain. The root mean squared fluctuation (RMSF) in the bottom panel was calculated from the three volume sub-classes derived from apoE and holoE cryo-EM data, respectively.

Biochemical studies indicate that rnpA promotes RNase P activity by enhancing binding affinity to pre-tRNA substrate^8,9^, but lack clear structural evidence to support this notion and the underlying mechanism. Our structures reveal that the interaction between rnpA and RPR is driven by hydrophobic and electrostatic interactions between two patches of positively charged rnpA residues and a region of the phosphate backbone of RPR with remarkable shape complementarity (Fig. 2a and 2b). Specifically, the sequence-conserved residues K53, K61, R62, and R65 in Helix 2 of rnpA interact directly with the RPR backbone phosphates of A336, U337, and A387 (Fig. 2b). The specificity is enforced through a π – π stacking interaction between the side chain of F13 in Helix 1 of rnpA and A334 of RPR (Fig. 2a, **upper right panel**). Notably, a large conformational change in RPR upon the binding of rnpA is observed in P19, one of the peripheral structural elements in the C-domain directly adjacent to the rnpA-binding site. The displacement of P19 is as large as 20 Å (Fig. 2c), caused by a bend at the coaxial stacking interface between P19 and P2, the latter of which is part of the rnpA binding site (Fig. 2c, **right panel**). Such discrete movements of P19 and other structural elements in the RPR S-domain, P9-10 and P12, are larger in apoE, and are constrained in holoE by rnpA (Fig. 2d **and Extended Data Fig. 2**) to reduce conformational heterogeneity of RPR. Thus, the RPR-rnpA interaction prepays the entropy penalty for engaging the pre-tRNA substrate.

### Substrate recognition by the RNase P holoenzyme

**Fig. 3 |:**
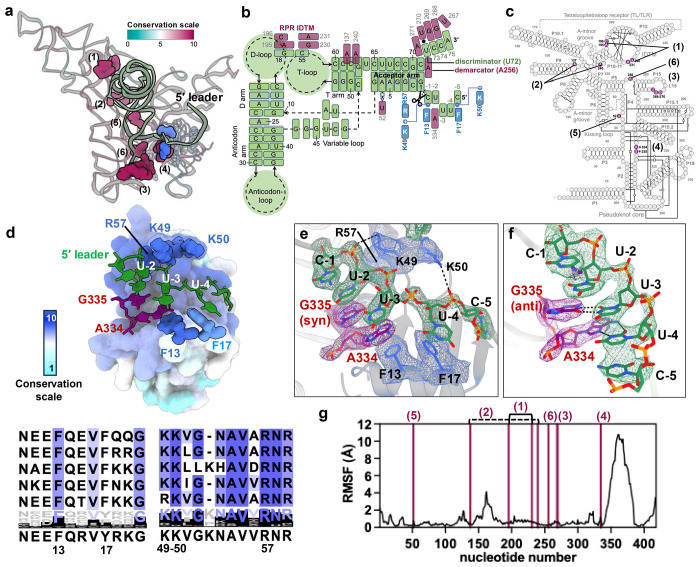
The six toeholds in RNase P holoenzyme (holoE) for pre-tRNA substrate recognition. **a**, Sequence conservation mapping on the structure of holoE in complex with pre-tRNA. Residues involved in the six toeholds are rendered in purple (RPR) and blue (rnpA) molecular surfaces. **b**, Secondary structure schematic of pre-tRNA (green), showing the toehold interactions with RPR (purple) and rnpA (blue) residues that comprise the substrate-binding interface. **c**, Secondary structure schematic of RPR showing the locations of all RPR toehold residues (purple). **d**, RnpA interaction with the pre-tRNA 5′ leader (green) and nucleotides G335, A334 of RPR (purple). The color-rendered surface of rnpA is scaled according to the sequence conservation shown below. **e**, Network of interactions involved in the recognition of the pre-tRNA 5′ leader (green) by RPR (purple) and rnpA (blue). The nucleobase side of the 5′ leader binds exclusively through hydrophobic interactions: RPR G335 with 5′ leader U-2, a triple base-stacking of rnpA F13, RPR A334, and 5′ leader U-3; and rnpA F17 with 5′ leader U-4. On the opposite side of the 5′ leader, the phosphate backbone is held by electrostatic interactions with rnpA K49, K50, and R57. **f**, Mode of 5′ leader recognition in the absence of rnpA, as observed in the structure of RPR pre-tRNA complex (apoES), which involves only two base pairs between RPR nucleobases A334 and G335 and pre-tRNA 5′-leader residues U-3 and U-2, respectively. Minimal hydrophobic stacking is achieved internally by adopting a quasi-helical structure. **g**, Root-mean-square fluctuation (RMSF) per residue of RPR, showing minimal changes in the six toeholds (purple), derived from 9 RNase P structures in complex with pre-tRNA substrate under various conditions, including 4 apoES structures and the 5 holoES structures in this study.

Pre-tRNA substrate recognition by holoE is achieved *via* six toeholds, one of which involves rnpA (Fig. 3a and 3b). The largest RPR conformational changes observed upon substrate binding occur in the IDTM and L15 regions, which interact with both the D- and T-loops in the pre-tRNA elbow region and the 3′-CCA trailer, respectively (Fig. 1d). Similar structural changes are observed in RPR complexed with mat-tRNA (Fig. 1d, **middle panel**), suggesting no major conformational transition upon release of the 5′ leader after the cleavage. Notably, RPR selectivity for pre-tRNA substrate and its cleavage-site specificity are achieved *via* the concerted interactions of the six toeholds, all of which involve residues that are not base-paired but sequence-conserved (Fig. 3c).

The 5′ leader up to U-4 is stabilized through electrostatic and hydrophobic stacking interactions (Fig. 3d). The hydrophobic stacking involves all three components of holoES (RPR, rnpA, and pre-tRNA 5′ leader): A334, G335 (RPR), U-2, U-3, U-4 (pre-tRNA 5′ leader), and F13, F17 (rnpA) (Fig. 3e). One of these interactions is a triple ring-stack among nucleobases A334 (RPR) and U-3 (pre-tRNA 5′ leader), and the phenyl ring of F13. The interaction with the 5′ leader is far more extensive in holoES as compared to apoES because of the presence of the rnpA, resulting in a different mode of binding (**Extended Data Fig. 4a and 4b**) and a 12% increase in contact surface. In apoES, this toehold is limited to base-pairing between U-4 and U-3 with RPR residues A334 and G335 (*anti*-conformation), respectively, forming a helical configuration (Fig. 3f). In holoES, the helical structure of the 5′ leader is not observed. Instead, the base-pairing interactions involving U-4 and U-3 are replaced with base-stacking interactions. RPR G335 (*syn*) is stacked between tRNA U-2 and U-3; A334 is stacked between U-3 and rnpA F13; and U-4 is stacked with F17 (Fig. 3e). On the opposite side of the 5′ leader strand, the phosphate backbone is stabilized through electrostatic interactions with the sidechains of rnpA residues K49, K50, and R57. The protein-enhanced interaction with the 5′ leader aids in catalysis by anchoring the cleavage site in place (lower activation energy). Thus, rnpA’s extensive interaction with the 5′ leader selectively enhances the affinity of holoE for pre-tRNA over mat-tRNA, consistent with the biochemical studies^7,8^. Importantly, no pre-tRNA nucleobases are in contact with rnpA *via* any specific hydrogen bonding, suggesting that the enhancing role of rnpA is sequence-independent.

In summary, the rnpA acts like a double-sided adhesive tape, with one side interacting with RPR to reduce its conformational flexibility (Fig. 2d), and the other directly engaging the pre-tRNA 5′ leader to enhance substrate-binding affinity. Importantly, among the apoES and holoES structures presented, the root mean square fluctuation (RMSF) of five out of the six toeholds (excluding the 5′ leader toehold) is about 1 Å (Fig. 3g **and Extended Fig. Data 4c-g**). This indicates that substrate recognition is self-contained within RPR, and that rnpA enhancement of pre-tRNA binding affinity is achieved not by structural rearrangement in the RPR but by strengthening one of the six pre-existing toeholds.

Interestingly, in the cryo-EM volumes of the holoES complex, we serendipitously observed a part of a second pre-tRNA molecule, whose 5′-leader was annealed to that of the first pre-tRNA *via* sequence complementarity in the region (−14 to −6) that extends beyond the RPR binding interface (**Extended Data Fig. 5a and 5b**). To ensure that the structure of the complex was not distorted due to the presence of the second pre-tRNA, we designed two pre-tRNAs that either prevented annealing (non-complementary) or promoted self-annealing (loop-back) in this upstream region. The holoES complex structures with either one of the pre-tRNA variants showed complete agreement with the 5′-leader conformation (up to −5) in the cognate pre-tRNA complex structure (**Extended Data Fig. 5c**).

### Mapping divalent metal ions

**Fig. 4 |:**
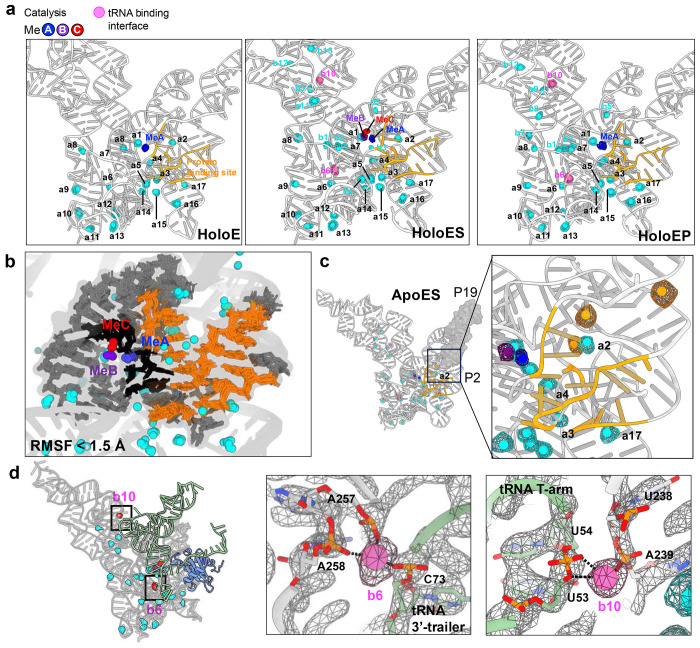
Divalent metal ions observed in each of the three catalytic states of RNase P holoenzyme (holoE, holoES, and holoEP). **a**, Overall divalent metal-ion landscapes in the holoE, holoES, and holoEP structures, colored and labeled according to their occurrence and functional roles: “a” (observed in all states); “b” (observed only in certain states); magenta (observed only in the presence of pre- or mat-tRNA); blue (catalytic metal ion A, observed in all states); purple (catalytic metal ion B) and red (catalytic metal ion C), observed only in holoES. **b**, Superposition of the RPR core of all structures determined in this and our previous study, illustrating the conservation of the metal-ion framework involved in stabilization of the structurally invariant regions consisting of catalytic nucleotides (black) and the rnpA protein binding site (orange). **c**, Three metal ions (yellow) observed in apoES are displaced upon rnpA binding. **d**, Ions b6 and b10 (magenta) stabilize the tRNA 3′-CCA trailer and T-arm, respectively, observed in holoES and holoEP complexes. RPR, rnpA, and pre-tRNA are colored in gray, blue, and green, respectively.

The resolution of the cryo-EM volumes presented in this study allows us to unambiguously observe the density of divalent metal ions (**Extended Data Fig. 2a**). We examined the coordinated metal ions of holoE and its complexes with pre-tRNA and mat-tRNA under various cation conditions (Fig. 4a **and Extended Data Fig. 2a**). For this analysis, we include only those that were visible in the cryo-EM volumes at a contour level ≥10 standard deviations (σ) and with a Q-score ≥0.7 (**Extended Data Fig. 2b**), all of which are found in the regions with a local resolution better than 2.8 Å (Fig. 4d, **Extended Data Figs. 1 and 2**). This resulted in a total of 33 unique metal ions among all 8 structures (**Extended Data Fig. 2**). Notably, these ions are located mostly in the RPR C-domain, where the structure is most rigid and well resolved (**Extended Data Figs. 1 and 2**). These metal ions were then classified into three groups. Group “a” ions (a1 to a17) are observed in all states (Fig. 4a, **cyan circles**); group “b” ions (b1 to b13) are only in certain states; and three metal ions, denoted MeA, MeB, and MeC, are the catalytic ions. Alignment of all RNase P structures in various enzymatic or ionic states shows that most of the metal ions, not just the catalytic ions, reside within the structurally invariant region of RPR (Fig. 4b). Three metal ions around the a2 ion at the junction of P2 and P19 observed in apo RPR structures are displaced upon rnpA binding (Fig. 4c, **right panel**), resulting in a conformational change in P19 (Fig. 2c). Many metal ions (b1 to b13) are seen only in the presence of tRNA (holoES or holoEP), all of which are involved in substrate binding (Fig. 4d). In particular, ions b6 and b10, located at the RPR-substrate interface (Fig. 4d), are uniquely important for stabilization of holoES, as they form several direct coordinate bonds with RPR and C73 and U54 of the tRNA 3′ trailer and T-arm, respectively (Fig. 4d, **inset panels**).

### Three-metal-ion mechanism of RNase P holoenzyme

**Fig. 5 |:**
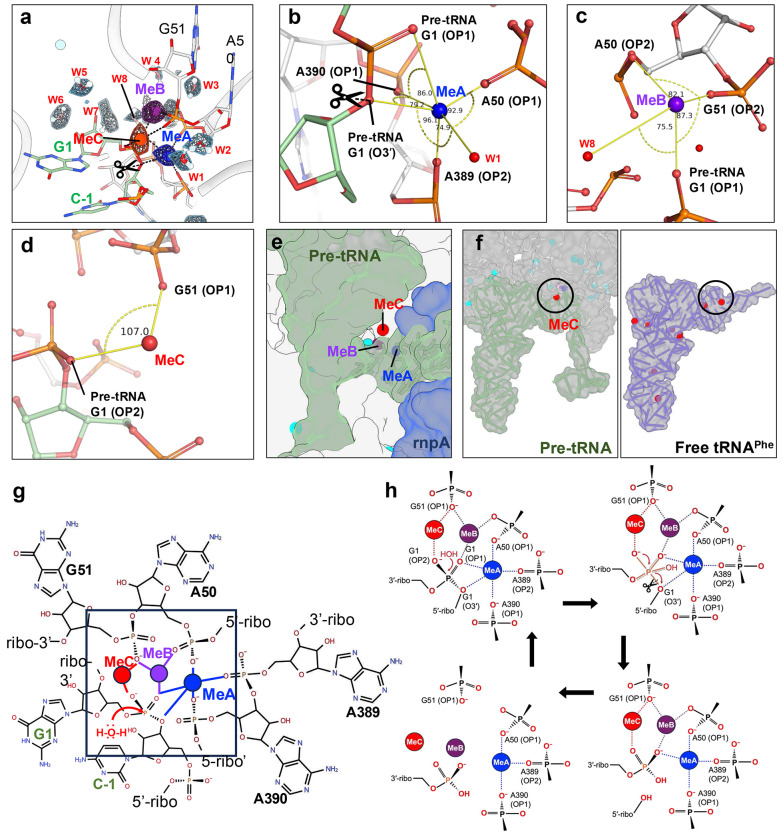
Active-site coordination and a three-metal-ion catalytic mechanism **a**, Three metal ions, MeA (blue), MeB (purple), and MeC (red), and ordered water molecules observed in the catalytic site of the holoES structure, indicating a three-metal-ion catalytic mechanism. MeA is observed in all three states (holoE, holoES, holoEP), whereas MeB and MeC are observed only in holoES. The cryo-EM density map for the ions and water molecules is shown in mesh, contoured at 10 standard deviations (***σ***). **b**, MeA (blue) coordination of RPR A50 (OP1), A389 (OP2), A390 (OP1), pre-tRNA G1 (OP1 and O3′), and a water molecule (W1), as observed in the structure of holoES. W1 may represent the reaction nucleophile. **c**, MeB (purple) coordination of RPR A50 (OP2), G51 (OP2), pre-tRNA G1 (OP1), and a water molecule (W8). **d**, MeC (red) coordination of RPR G51 (OP1) and pre-tRNA G1 (OP1). **e**, The cavity formed by the surfaces of pre-tRNA and rnpA, which reduces the solvent accessibility of the three catalytic metal ions (MeA, MeB, and MeC). **f**, MeC ion position relative to pre-tRNA substrate in holoES structure. **g**, Divalent metal ions observed in the yeast tRNA^Phe^ structure^27^, in the same orientation as the left panel. **h**, Model of the phosphodiester hydrolysis reaction by the RNase P holoenzyme *via* a three-metal-ion mechanism, based on the structures presented in this work. The complete reaction cycle: pentacovalent transition-state intermediate formation, stabilized by MeA, MeB, and MeC (top-left), breaking of the phosphodiester bond (top-right), dissociation of MeB and MeC along with 3′ and 5′ products (bottom-right), and return to the holoE initiation state (bottom-left).

The presence of MeA throughout the entire catalytic cycle at different concentrations of divalent metal ions (Fig. 4a **and Extended Data Figs. 6–7**) indicates that neither pre-tRNA nor rnpA protein is required for proper coordination of this catalytic metal ion. MeB, on the other hand, is only observed when pre-tRNA substrate is bound (Fig. 4a). In the holoES structures, we observed a third metal ion (MeC) (Fig. 5a), which is present only when both protein and substrate are bound, demonstrating that the RNase P holoE uses three metal ions for catalysis, in contrast to the long-proposed two-metal-ion mechanism^13^. The presence of MeC in holoES, therefore, provides a third enhancing role for rnpA by facilitating the recruitment of a third active-site metal ion (MeC). Moreover, the absence of MeB and MeC in holoEP suggests that these two ions may stabilize the product 5′ leader strand—the leaving group of the nucleophilic substitution reaction (Fig. 4a, **far-right panel**).

Five oxygen atoms tightly coordinate metal ion A (MeA) with a pentagonal bipyramidal coordination geometry of Ca^2+^. The equatorial plane is formed by RPR pseudoknot nucleotide backbone phosphates A50(OP1) and A389(OP2), and pre-tRNA G1(O3′). The pre-tRNA G1(OP1) and A390(OP1) occupy two axial positions. A total of eight water molecules could be identified in the active site, two of which are directly involved in the catalytic metal-ion coordination network (Fig. 5a **and Extended Data Fig. 7**). One of these waters (W1) is directly coordinated by MeA and is the closest water to the G1 scissile phosphorus (4.0 Å) (Fig. 5b). It is plausible that the coordination geometry of a Mg^2+^ in the position on MeA may place W1 within reaction distance, therefore making this water the potential nucleophile. MeB is coordinated by pre-tRNA G1 (OP1), G51 (OP2) and A50 (OP2) of RPR, and a water molecule (W8) (Fig. 5c). And the third catalytic metal ion, MeC, is coordinated by RPR G51 (OP1) and pre-tRNA G1 (OP2) (Fig. 5d). Though MeC exhibits only two coordinate bonds, as compared to MeA (6-bonds), and MeB (4-bonds) (Fig. 5b and 5c), and is much more solvent accessible (Fig. 5e), it is observed at physiological (1 mM) metal-ion concentration (**Extended Data Fig. 6**).

MeC is of unique functional importance in the context of holoE and, along with rnpA, could contribute to the increased activity of holoE relative to apoE. Even though rnpA has no direct interactions with the catalytic metal ions, its presence is required for MeC recruitment. Based on its position, the enhancing role of MeC is likely two-fold: 1) adding to the coordination of the scissile phosphate, and 2) further stabilizing the reaction leaving group. To further investigate MeC, we compared the pre-tRNA structure in holoES (Fig. 5f, **left panel**) to the yeast tRNA^Phe^ crystal structure, which contains eight metal ions^27^ (Fig. 5f, **right panel**). The two metal ions (circled area) located in the major groove of the acceptor arm of tRNA^Phe^ (Fig. 5f, **right panel**) are 4.0-6.7 Å away from the MeC position in holoES. It is plausible, therefore, that MeC may be supplied by the pre-tRNA substrate, and it becomes properly positioned in the catalytic site upon rnpA-mediated coordination with RPR. In summary, the structural evidence of the metal ions found in the the catalytic site of the holoES provide a basis for three-metal-ion mechanism for RNase P holoenzyme (Fig. 5g). In the presence of substrate, MeA directly coordinates the O3′ atom of the phosphodiester scissile bond of pre-tRNA to stabilize the cleavage site (Fig. 5b). Since MeA is always present, and RNase P activity is dependent on pH, Mg^2+^ concentration^12,28^, and rnpA^7–9^, the coordination of MeB and MeC in the catalytic site is thus a rate-limiting step in the reaction cycle (Fig. 5h). The three catalytic metals work synergistically with both tRNA (G1 O3′; G1 OP1; G1 OP2) and RPR (A389 OP2; A390 OP1) backbone to form the trigonal-bipyramidal, pentacoordinate phosphorane transition state (Fig. 5h, **upper-left panel**), consistent with the general phosphoryl transfer reactions of other enzyme systems^29,30^. After cleavage, both MeB and MeC dissociate from the catalytic site, along with mat-tRNA and the 5′ leader (Fig. 5h, **upper-right to bottom-left panels**).

### Role of tetraloop/tetraloop-receptor interaction

**Fig. 6 |:**
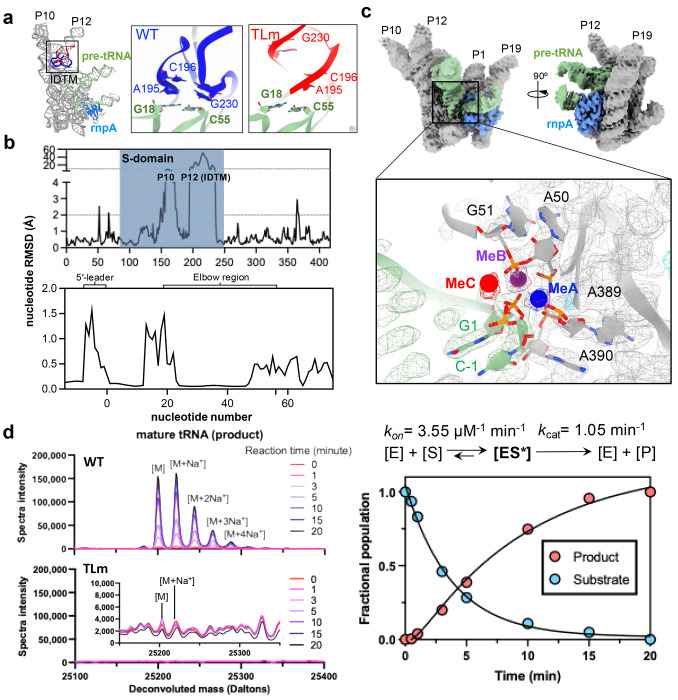
Role of the tetraloop/tetraloop-receptor (TL/TLR) interaction on RNase P Holoenzyme structure and activity. **a**, Structural comparison of wild-type (WT) and tetraloop mutant (TLm) holoE, with insets (right two panels) showing how the loss of the TL/TLR interaction in TLm-holoE results in the rearrangement of the IDTM into an unstable helical configuration. **b**, Comparative analysis of WT and TLm-holoES structures via per-nucleotide root-mean-square deviation (RMSD) of RPR (top) and pre-tRNA (bottom). **c**, Structure of TLm-holoES and the three catalytic metal ions observed in the active site (inset panel). **d**, Time-course experiments and reaction kinetics of the hydrolysis of cognate pre-tRNA^Gly^ by WT-holoE (top-left) and TLm-holoE (bottom-left) by monitoring the mass distribution of mature tRNA product formation, conducted using liquid chromatography quantitative mass spectrometry, showing that TLm nearly or completely abolishes ribozyme activity. For clarity, the inset panel is the magnification of the mass distribution of the mature tRNA product by TLm-holoE. The kinetic reaction scheme and rate constants for pre-tRNA hydrolysis were determined from initial-rate measurements using the Michaelis–Menten steady-state model (right panel).

The RPR S-domain includes two key structural elements: the tetraloop/tetraloop-receptor (TL/TLR) interaction and the interdigitated double T-loop motif (IDTM). While IDTM serves as one of the six toeholds in pre-tRNA recognition and binding, the role of TL/TLR interaction in terms of how this interaction couples with IDTM formation is unknown. For this reason, we determined the structure of the ternary complex by replacing the tetraloop (GAAA) with “UUUU” (TLm) in P12, abolishing the TL/TLR interaction. The mutation resulted in abolishing the TL/TLR interaction as shown in the structure, where P10 and P12 separate leading to improperly folded IDTM, with the remainder of the RPR structure unchanged in the TLm-holoE ternary complex compared with the wilde-type (Fig. 6, **and and Supplementary Video 1**), but the remaining structure of the mutant complex is nearly identical to the wild-type holoES complex (RMSD <1.5 Å), including the presence of all three catalytic metal ions (Fig. 6c, **bottom panel; Supplementary Fig. 6**). The resulting changes in the S-domain include regions that form key interactions with the pre-tRNA 5′-leader and elbow regions (Fig. 6b, **bottom panel**) as well as much lower substrate occupancy observed as compared to wild-type RNase P, where only 14% out of total particles can be used to build the TLm holoES structure (**Supplementary Figs. 4 and 6**). Using quantitative liquid chromatography mass spectrometry, we demonstrate that the TLm nearly or completely abolishes cleavage activity by holoE (Fig. 6d). Altogether, we show that IDTM is important for substrate recognition and binding (Fig. 3). The proper folding of this structural element is dependent on the TL/TLR interaction within the RNA component. In contrast, the IDTM conformation (Kink-turn motif) in the human nuclear RNase P RNA is stabilized by its protein components, Rpp21, Rpp29, and Rpp38, within the RNase P ribonucleoprotein complex^31^.

## Discussion

The structures of holoE, holoES, and holoEP presented in this work illustrate that rnpA plays three functional roles. First, the comparison of holoE and apoE structures shows that rnpA binding results in a large shift in P19 that is favorable for pre-tRNA binding (Fig. 2c and 2d). Second, rnpA works together with RPR to accommodate the 5′ leader through an extensive network of hydrophobic stacking and electrostatic interactions to secure substrate (Fig. 3d–f), forming and maintaining the three-metal-ion active-site coordination framework (Fig. 4a). Third, consistent with previous findings, the interaction with the 5′ leader selectively promotes binding of pre-tRNA over mat-tRNA, and facilitates product release^7^. Together, these findings explain the protein’s role in enhancing activity and why the holo RNase P ribozyme is more efficient, despite the substrate and active site being in the proper configuration.

The three–metal-ion model was proposed based on biochemical studies of the protein-free Group I intron^32^, but structural data support a two–metal–ion model^33,34^. Our present study demonstrates the presence of three metal ions in the structure of the *Gst* RNase P holoenzyme–pre-tRNA complex. These three metal ions adopt an arrangement similar to the catalytic metal-ion triad of human DNA polymerase^22^ (**Extended Data Fig. 7**), suggesting a case of convergent evolution of RNA and protein metalloenzymes for catalysis of phosphoryl transfer reaction.

The bacterial RNase P holoenzyme structures in complex with pre-tRNAs in this study provide important insights into the roles and evolution of the RNase P protein components. Instead of employing a single protein component in bacterial RNase P that mainly stabilizes the leader sequence binding and the third metal ion, archaeal and eukaryotic RNase Ps function synergistically with 5 to 10 protein components^35,36^, four of which are protein orthologs across archaea and eukaryotes, namely Pop5, Rpp21, Rpp29 (Pop4), and Rpp30. A comparative structural analysis among bacterial, archaeal, and eukaryotic RNase Ps showed that Rpp21, Rpp29 (Pop4), and Rpp30 create an additional contact interface, mostly on the tRNA anticodon arm, for the tRNA substrate recognition (**Extended Data Fig. 8a**), which could suggest some differences in how bacterial, archaeal, and eukaryotic RNase Ps recognize the pre-tRNA substrate^18,19^. Such expansion and elaboration in the binding surfaces for substrate recognition through the acquisition of accessory protein components may have provided a pathway to subject archaeal and eukaryotic RNase Ps to additional layers of regulation^37^. Strikingly, even though none of the rnpA protein analogs are found in archaeal and eukaryotic RNase Ps, our structural comparison indicates that the Pop5 protein in archaeal and eukaryotic RNase Ps, which shares the same composition of positively charged surface in proximity to the 5′ leader of pre-tRNA, may serve a functionally similar role as rnpA (**Extended Data Fig. 8b**).

## Methods

### RNase P RNA sample preparation

RNase P RNA was transcribed *in vitro* in transcription buffer (20 mM potassium-HEPES buffer, pH 7.5, 25 mM MgCl_2_, 1 mM Dithiothreitol) for 3 hours with recombinant T7 phage RNA polymerase and double-stranded DNA template amplified by polymerase chain reaction (PCR) from a full-synthesized plasmid pUC18. This plasmid encodes the full-length RNase P RNA sequence from bacterial strain *Geobacillus stearothermophilus* (GenBank access number: M19021.1) with an upstream T7 RNA polymerase promoter sequence, GGATCCAGCTCGAAATTAATACGACTCACTATA. After *in vitro* transcription (IVT), the magnesium pyrophosphate precipitation was removed by centrifugation at a spin rate of 13,000 rpm for 10 min using a high-speed benchtop centrifuge. The final concentration of 200 mM sodium chloride (NaCl) was then added to the IVT solution for overnight refolding at 4°C before further purification. The refolded RNA was subjected to a Fast Protein Liquid Chromatography (FPLC) apparatus (GE HealthCare ÄKTA^™^ pure) and purified by a nondenatured method^38^ through size-exclusion chromatography (SEC) column (HiLoad^™^ 16/600 Superdex^™^ 200 increase). The column was equilibrated with the SEC elution buffer (25 mM Tris, pH 7.5, 100 mM NaCl, 5 mM CaCl_2_ or MgCl_2_) before purification, and the monomeric RNase P RNA molecules were eluted with a flow rate of 0.5 mL/min and separated from aggregation species. Eluting RNase P RNA was detected by absorbance at 280 and 260 nm; peak fractions were collected based on the SEC chromatogram and stored at 4°C.

### Mutagenesis for RNase P tetraloop mutation

The DNA template of the RNase P tetraloop mutant was generated by site-directed mutagenesis. The two primers designed for mutagenic PCR are GGAGCTCTAAGGTTTTCCTTAGAGGTGG (sense) and CCACCTCTAAGGAAAACCTTAGAGCTCC (anti-sense), for the replacement of the GAAA tetraloop with four consecutive pyrimidine bases, UUUU, which prevents the tetraloop/tetraloop receptor interaction. The mutagenesis PCR was performed using high-fidelity *Taq* DNA polymerase (Platinum^®^, Invitrogen Thermo Fisher Scientific), with a 95°C denaturation step for 1 minute, followed by 30 amplification loop-back cycles (95°C denaturation for 40 seconds, 62°C annealing for 40 seconds, and 72°C elongation for 3 minutes). The PCR product was transformed into TOP10 *E. coli* competent cells (Invitrogen, Thermo Fisher Scientific) for subsequent colony screening. The DNA sequence of the RNase P tetraloop mutation was confirmed by capillary electrophoresis DNA sequencing (Quintarabio, USA).

### Expression and purification of *Geobacillus stearothermophilus* RNase P protein component rnpA

*Geobacillus stearothermophilus* (*Gst*) RNase P protein subunit, rnpA (NCBI reference sequence: WP_049624131.1), was cloned into the Gateway^™^ pDEST^™^ vector *via* Gateway Recombination Cloning. The recombinant rnpA protein contains an amino-terminal hexa-histidine affinity tag (N-terminal his-tag) and tobacco etch virus (TEV) protease cleavage sequence between the his-tag and rnpA coding sequence.

The protein was expressed in *E. coli* strain BL21(DE3) induced with 0.5 mM isopropyl β-D-1-thiogalactopyranoside (IPTG) in rich media (Dynamite broth^39^) at 16 °C for 16 hours. The cell pellet of a 500-ml culture was lysed using a cell homogenizer (SPX APV homogenizer), followed by centrifugation at a speed of 6,000 × g for 30 minutes to remove the bacterial membrane debris. The rnpA protein with an N-terminal His-tag in the supernatant was purified using immobilized metal affinity chromatography (IMAC) with Cobalt-NTA agarose resins, followed by TEV-protease digestion to remove the His-tag. The rnpA after TEV protease digestion was subjected to Fast Protein Liquid Chromatography (FPLC) apparatus (GE HealthCare ÄKTA^™^ pure) and purified through size-exclusion chromatography (SEC) column (Superdex^®^ 75 Increase 10/300 GL) with the protein storage buffer (20 mM HEPES, pH 7.2, 300 mM NaCl, 0.5 mM TCEP). The amount of the purified rnpA protein was quantified using the Pierce BCA Protein Assay Kit (Thermo Fisher Pierce BCA kits), aliquoted, and kept at −80°C.

### Precursor and mature tRNA sample preparation

Bacterial precursor (pre-) tRNA^Gly^ DNA template harboring an upstream T7 RNA promoter, followed by a 14-nucleotide-long 5′-leader sequence and a 6-nucleotide-long 3′-trailer, CCAAUA, was fully synthesized by Ultramer^™^ service (Integrated DNA Technologies, USA). PCR generated a double-stranded DNA template for *in vitro* transcription to produce a 92-nucleotide-long pre-tRNA. Production of mature tRNA^Gly^ (mat-tRNA) that lacks the 5′-leader sequence follows the same expression scheme. The *in vitro* transcribed pre-tRNA^Gly^ and mat-tRNA were natively purified using a size-exclusion chromatography (SEC) column (HiLoad^™^ 16/600 Superdex^™^ 200 increase) with 1 mM Ca^2+^ or Mg^2+^ buffer (25 mM Tris-HCl pH 7.5, 100 mM NaCl, 1 mM CaCl_2_ or MgCl_2_), depending on the purpose of the experiments.

The non-complementary (nc) and loop-back (LB) pre-tRNA variants are designed to prevent the hybridization of the 5′-leader sequence (**Extended Data Fig. 5**). The 5′-leader of nc-pre-tRNA is GGCUCUUAACUUUC in place of the original 5′-leader sequence, which can prevent the 5′-complementary pairings between the two pre-tRNAs.

Following a similar rationale, a loop-back hairpin sequence, GGAUCCGGAUCCUUUUGGAUCCGGAUCCCUUUC, in the 5′-leader of LB-pre-tRNA forms an intramolecular hairpin, preventing the 5′-complementary pairings between the two pre-tRNAs.

### Enzymatic assay of 5′ maturation of precursor tRNA quantified by liquid chromatography-mass spectrometry

Before the assay, RNase P holoenzyme (holoE) was made by mixing apoenzyme and the rnpA protein component with the same molar ratio at 1 mM Mg^2+^ reaction buffer (25 mM Tris-HCl, pH 7.5, 100 mM NaCl, 1 mM MgCl_2_). The final concentration of holoE is 0.2 μM, with a holoE to pre-tRNA molar ratio of 1:10. The reactions were conducted at 37°C in the presence of 25 mM Tris-HCl, pH 7.5, 100 mM NaCl, and 5 mM MgCl_2_. For the time-course experiments of hydrolysis of pre-tRNA^Gly^ mediated by RNase P, the reaction at each time point was stopped by adding the quench buffer (25 mM Tris-HCl, pH 7.5, 10 mM EDTA) and incubating at 4°C before quantitative analysis *via* liquid chromatography-mass spectrometry (Q–LC–MS). To confirm the 5′ cleavage fidelity by apoE and holoE, the 5′-leader products after holo and apoE digestion were isolated and buffer-exchanged in RNase-free double-distilled water solution to remove the salt in the buffer before Q–LC–MS analysis. The result is shown in **Supplementary Fig. 1**.

Q–LC–MS time-course experiments were performed on a 6520 Accurate-Mass Q–TOF LC/MS system (Agilent Technologies, Inc.) equipped with a dual electrospray ionization (ESI) source, operated in positive-ion mode. Samples included 2 *μ*M pre-tRNA and the non-complementary (nc) pre-tRNA digested by RNase P holoenzyme at various time points (0, 1, 3, 5, 10, and 20 minutes). Acetonitrile was added to all samples to a final concentration of 10%. The 20 *μ*l supernatant was transferred to glass injection vials for Q–LC–MS analysis. Q–LC–MS was performed with a TSQ Quantiva triple quadrupole mass spectrometer (Thermo Fisher Scientific) operating in selected reaction monitoring mode with positive ESI and with a Shimadzu 20AC-XR system using a 2.1 × 100 mm^2^, Charity 2.6 μm Oligo-MS 100A Phenomenex^®^ C18 HPLC column (Part number: 00D-4479-AN). Data acquisition and analysis were performed using a Mass Hunter Workstation (v.B.06.01). Mass Hunter Qualitative Analysis software (v.B.07.00) with BioConfirm Workflow was used to present and deconvolute mass spectra. The distributions of different ionization states of the deconvoluted mass were shown in **Supplementary Fig. 2**.

The deconvoluted mass distributions of pre-tRNA substrate and mat-tRNA product at the different time points served as numerical matrices, which are subject to singular value decomposition (SVD) analysis using MATLAB (R2022a) to extract the significant singular value components of the relative abundance of the mass distribution for the following kinetic analysis. The deconvoluted mass distributions from SVD were used to derive the association rate of enzyme-substrate complex formation, $k_{\text{on}}$, and catalytic constant, $k_{\text{cat}}$, based on a consecutive, irreversible first-order reaction scheme, shown as follows.

Equation (1) $$\text{[holoE] + [pre-tRNA]}\overset{k_{on}}{\to }\text{[holoES]}\overset{k_{\text{cat}}\text{​}}{\to }\text{[holoE] + [mat-tRNA]}$$

For this mechanism, the initial substrate concentration, [pre-tRNA]_0_ is much greater than the total enzyme concentration [holoE], so that the initial rate of [holoES] is

Equation (2) $$\frac{-d[\text{holoES}]}{dt}=k_{on}[\text{holoE}]-\left(k_{on}+k_{cat}\right)[\text{holoES}]$$

Equation (2) can be integrated to yield a time-dependent Equation (3):

Equation (3) $$[\text{holoES}]=\frac{k_{\text{on}}[\text{holoE}]}{k_{\text{on}}+k_{\text{cat}}}\left[1-e^{-\left(k_{\text{on}}+k_{\text{cat}}\right)t}\right]$$

Together with the initial rate of [mat-tRNA] product,

Equation (4) $$\frac{d[\text{mat-tRNA}]}{dt}=k_{\text{cat}}[\text{holoES}]$$

The time dependence of product formation and depletion of substrate can be written as follows:

Equation (5) $$[\mathrm{mat}-\mathrm{tRNA}]=\frac{k_{\text{on}}k_{\text{cat}}[\mathrm{holoE}]t}{k_{\text{on}}+k_{\text{cat}}}+\frac{k_{\text{on}}k_{\text{cat}}[\mathrm{holoE}]}{{\left(k_{\text{on}}+k_{\text{cat}}\right)}^{2}}\left[e^{-\left(k_{\text{on}}+k_{\text{cat}}\right)t}-1\right]$$

Equation (6) $$\left[\text{pre}-\text{tRNA}]={\left[\text{pre}-\text{tRNA}\right]}_{0}-[\text{mat}-\text{tRNA}\right]$$

The nonlinear regression fit using Equation (5) for the kinetic rates, $k_{\text{on}}$ and $k_{\text{cat}}$ were conducted by GraphPad Prism (10.6.1) and summarized in **Supplementary Table 4**.

### Cryo-EM sample preparation and data acquisition

The RNase P holoenzyme (holoE)-to-pre-tRNA molar ratio for holoES complex formation is 1:3 at a final concentration of 4 mg/mL; the complex was formed by incubating for 30 minutes at 4°C in a buffer containing 25 mM Tris, pH 7.5, 100 mM NaCl, and 5 mM CaCl_2_. RNase P holoE to mat-tRNA molar ratio for the holoEP complex formation is 1:4; the complex was formed with 30-minute incubation at 4°C in the same buffer condition.

Quantifoil Au grids (R1.2/1.3, 300 mesh) were glow-discharged on each side for 60 seconds at 25 mA. Four microliters of sample, ranging from 2.8 to 4 mg/mL, were applied to the carbon side of the grid. After 10 seconds, the grids were blotted for 3 seconds at 100% humidity at 6 °C and plunge-frozen in liquid ethane using a Vitrobot Mark IV (Thermo Fisher Scientific). All data were collected using a Talos Arctica G2 (200 keV) equipped with an X-FEG, Gatan BioQuantum imaging filter. The movies were recorded in super-resolution mode using a Gatan K3 direct electron detector at a magnification of 100K (pixel size of 0.81 Å), with 2.5-second exposures at a dose rate of ~15 e^−^/Å^2^/s, resulting in a total dose of ~57 e^−^/Å^2^.

### Cryo-EM data processing

All data processing was performed in cryoSPARC^40^. Movies were patch-motion-corrected with a Fourier-crop factor of 0.5, followed by patch Contrast Transfer Function (CTF) estimation. Curated micrographs were denoised, and particles were selected using a template of 2D classes. The particles were extracted and binned to a pixel size ranging from 2.4 to 3.2 Å and subjected to several iterative rounds of reference-free 2-dimensional (2D) classification. The 2D-cleaned particles were used to generate 1 to 3 *ab initio* volumes, the best of which was used for non-uniform refinement to align the full stack of particles for 3-dimensional (3D) reconstruction. The particles were then pruned in 3D using one or more iterative rounds of heterogeneous refinement against the refined volume and three or more decoy volumes. The cleaned particle stack was reextracted with a box size of 512 pixels and a pixel size of 1.04 Å and was split into 2–3 subclasses by 3D classification (without alignment). In most cases, the subclasses only showed significant fluctuations in P19 and, in part, the substrate specificity (S) domain, which could be further subclassified (**Supplementary Fig. 3**). For simplicity, however, the particle stacks in these cases were kept aligned to a single consensus volume. For all structures complexed with tRNA, the particles containing precursor tRNA (pre-tRNA) or mature tRNA (mat-tRNA) were sorted from those without by 3D classification using a focus mask around the tRNA (**Supplementary Fig. 4**). In the case of the holoES complex containing the loop-back pre-tRNA, the particles were classified further based on the two tilt angles of the anticodon arm of pre-tRNA (**Supplementary Fig. 5**).

The RNase P RNA tetraloop mutant (holoE-TLm), which lacks the tetraloop/tetraloop-receptor interaction in the S-domain, appears to be more structurally heterogeneous than WT. In this case, 3D classification was applied to subclassify the particles into three different conformer structures, where the two structural elements of the S-domain, P10 and P12, adopt different positions (**Supplementary Fig. 6**). As the TLm has much lower affinity for pre-tRNA, holoE-TLm and holoES-TLm complex structures were derived from a single data set after sorting by 3D classification using a focus mask around the pre-tRNA.

The final particle stacks in the aforementioned cases and their corresponding exposures were clustered and split into exposure groups according to beam shift. These particles were then subjected to further non-uniform refinement, including optimization of the per-particle scale, defocus, and per-exposure-group CTF parameters at each iteration. The resulting particle stacks, volumes, and masks were then used for reference-based motion correction (RBMC). In some cases, further pruning could be achieved by heterogeneous refinement (with decoys) and/or 3D classification after RBMC. In these cases, the improved particle stacks and volumes were further refined and used for a subsequent round of RBMC. The particle stacks from RBMC were used for a final round of non-uniform refinement, followed by local refinement using the output mask.

### Model building and refinement

The initial structural model of *Gst* RNase P RNA component (RPR) was built using RNA homology modeling^41^ with the two crystal structures (PDB IDs: 2A64^42^ and 1NBS^43^). The full-length *Gst* RPR structure was fitted into the cryo-EM volume, refined, and regularized in real space using Coot (0.9.8.96)^44^. After model fitting and regularization, SimRNA^45^ was used to minimize the global and local energies of the RNA structures.

To build *Gst* RPR in complex with its tRNA substrate and protein component, the structures of precursor tRNA substrates and mature tRNA products were built with an initial structural model of the bacterial tRNA^Gly^ (PDB ID: 4MGN^46^, chain B). The initial structural model of the *Gst*-RNase P protein component rnpA was generated by AlphaFold2^47^ by providing the primary sequence of rnpA (NCBI reference sequence: WP_049624131.1). The missing regions of both tRNA^Gly^ substrate and protein were modeled and real-space-refined together with *Gst* RPR structure using Coot (0.9.8.96)^44^. The final models of *Gst* RPR and other enzymatic states were refined and validated against their respective unsharpened cryo-EM volumes using Phenix (1.21.2)^48^. Structure and validation statistics, including correlation coefficients (C.C.), Fourier shell correlation (FSC) map resolution, all-atom clash scores, and other refinement parameters, are summarized in **Supplementary Tables 1 and 2**.

For building the divalent metal ions, we include only the metal ions whose cryo-EM maps were visible at a contour level ≥10 standard deviations (SD) (**Extended Data Fig. 2a**) with a cryo-EM resolvability Q-score^49^ ≥0.7 (**Extended Data Fig. 2b**). Given the coordination distances between metal ions and closest phosphate groups^50^, the directly coordinated metal ions and hydrated metal ions can be further defined (Fig. 4b and 4c). Given the buffer conditions for each structure, the only probable ions are Na^+^ and Ca^2+^/Mg^2+^. We analyzed metal ions using the CheckMyMetal program^51^ by assessing coordination geometry, contact, distance, B-factor, and occupancy of the metal ions in our structures (summarized in **Supplementary Table 3)**. Although we cannot completely rule out the possibility of Na^+^ in some ambiguous cases, given the saturating concentrations of divalent ions in the buffer and that ordered Na^+^ ions are rarely observed or reported in RNA structures, we then model them as divalent ions. All assigned metal ions in our structures exhibit high cryo-EM volume occupancy and Q-scores (**Extended Data Fig. 2**).

## Data Availability

Cryo-EM reconstructions and RNase P holoenzyme (holoE), holoenzyme-substrate complex (holoES), holoenzyme-product complex (holoEP), and TLm-holoenzyme-substrate complex (TLm-holoES) models have been deposited at the Electron Microscopy Data Bank (EMDB) at https://www.ebi.ac.uk/emdb/ and Protein Data Bank (PDB) at http://www.pdb.org, respectively. Accession numbers for EMDB are EMD-70941, EMD-70942, EMD-70943, EMD-70944, EMD-70945, EMD-70946, EMD-70947, EMD-70948, EMD-70997, EMD-70988, EMD-71000. Corresponding accession codes for PDB are 9OWR, 9OWS, 9OWT, 9OWU, 9OWV, 9OWW, 9OWX, 9OWY, 9OY5, 9OY6, 9OY7.
